# The Janet Abortive Treatment of Gonorrhea

**Published:** 1897-02

**Authors:** 


### Selections and Abstracts.

### THE JANET ABORTIVE TREATMENT OF GONORRHŒA.

In a second communication on this subject, published in the *Centralblatt fur innere Medicin* for October 10th, Dr. Berthold Goldberg, of Cologne, expresses his conviction that the early treatment of gonorrhœas by means of frequent injections of a solution of potassium permanganate is a trustworthy means of aborting the disease. He gives a table of fourteen cases, in all but one of which he succeeded in curing the affection within so short a time as to justify the use of the term abortion.

Six of the patients were suffering from a first attack of gonorrhœa, five were affected with a second attack, two had their third attack, and one was in his fourth. The treatment was begun in two days after the infection (twelve hours after the first symptom) in one case, in three days after the infection (twelve hours after first symptom), in two cases in six days after the infection (one day after first symptom) in two cases, in seven days after the infection (one day after the first symptom in one case and an unnoted length of time in the other) in two cases, in eight days after the infection (one day after first symptom in two cases and six days in one case) in three cases, in nine days after the infection (two days after the first symptom) in one case, in ten days after the infection (three and four days respectively after the first symptom) in two cases, in twelve days after the infection (seven days after the first symptom) in one case.

Five injections sufficed to effect a cure in one case, six in three cases, seven in one case, eight in one case, nine in one case, eleven in one case, twelve in one case, thirteen in two cases, fourteen in one case, and fifteen in one case, while about thirty were employed in the case which, although finally cured, in about a hundred days, was not aborted. In that case the patient went from the seventh to the tenth day without treatment. As a complication, he had follicular prostatitis. The only complication in any of the other cases was bacteriuria in one case, which did not interfere with a prompt cure. The deep urethra was involved and had to be irrigated in six of the cases. The gonococci disappeared in one day in two cases, in three days in two cases, in five days in one case, in six days in one case, in ten days in one case, in eleven days in two cases, in twelve days in one case, in fourteen days in one case, in about fourteen days in one case, and in about a hundred days in one case—the one that was not aborted. In one case this detail was not recorded, but in that case all traces of the disease had disappeared in four days. In almost all the cases the urine ceased to show any signs of a discharge coincidently with the disappearance of the gonococci, but in three of the cases not until after a period of from three to six days: the urine was examined at intervals of from two to a hundred days after the subsidence of the disease. All the patients were allowed to go about as usual during the treatment.

When it was practicable, Dr. Goldberg gave the injections himself twice a day, but several of the patients were unable to present themselves so often and consequently used the syringe themselves for a portion of the time, employing a solution of the strength of from 1 to 2,000 to 1 to 4,000 and injecting only the anterior urethra. Moreover, in several of the cases the treatment by the author himself had to be intermitted for as much as three days. All things considered, this seems to be a remarkably good showing for the Janet treatment.—*New York Medical Journal.*

